# Development and performance evaluation of a Bengali-adapted mHealth app for early detection of impaired visual acuity

**DOI:** 10.1016/j.heliyon.2024.e40853

**Published:** 2024-12-03

**Authors:** Monzurul Haque, Marzia Zaman, Ashraful Islam, Farhana Sarker, Nahid Ferdausi, Khondaker A. Mamun

**Affiliations:** a AIMS Lab, IRIIC, United International University, Dhaka, Bangladesh; b Center for Computational & Data Sciences, Independent University, Bangladesh, Dhaka, Bangladesh; c Department of Computer Science and Engineering, Independent University, Bangladesh, Dhaka, Bangladesh; d Department of Computer Science and Engineering, Southeast University, Dhaka, Bangladesh; e Gopalganj Eye Hospital and Training Institute, Gopalganj, Bangladesh; f Department of Computer Science and Engineering, United International University, Dhaka, Bangladesh; g CMED Health Limited, Dhaka, Bangladesh

**Keywords:** mHealth, Eye, Visual acuity, Vision problem, Near vision, Distant vision

## Abstract

This paper presents a comprehensive study on the development and performance evaluation of “*Dristi*,” a mobile health (mHealth) app designed to facilitate eye screening for impaired visual acuity (VA) in Bangladesh. Recognizing the critical importance of vision and the challenges posed by visual impairments, particularly in low-resource settings, this study explores an innovative solution to enhance eye care accessibility. The app, developed for Android devices, integrates features such as VA testing using adapted Bengali letters, color blindness tests, and eye health education, aiming to make eye care more user-friendly and accessible. Inspired by the Snellen chart, the app employs a simplified method for VA assessment, suitable for the constraints of mobile devices. The study involved a cross-sectional comparative design, conducted between June and September 2022, to clinically validate the app's feature of testing distant vision and near vision. Participants were recruited from one government eye hospital, one semi-private eye hospital, one high school, and a private clinic in Bangladesh, encompassing a diverse demographic. The validation study revealed promising results in terms of the app's accuracy, sensitivity, and specificity in both distant and near vision testing, although some variability was observed across different data sources. The study also identified a significant number of participants with diabetes and hypertension, common comorbidities affecting eye health. Overall, “*Dristi*” demonstrates potential as a valuable tool in the early detection and management of visual impairments in Bangladesh. Its development marks a significant step towards utilizing mHealth solutions to overcome healthcare barriers and improve eye care services in low-resource settings. Future directions include expanding the app's features, enhancing its integration with healthcare systems, and extending its reach to other similar contexts.

## 1 Introduction

Vision is a fundamental human sense, crucial for experiencing the beauty and details of the world and our surroundings. Unfortunately, the privilege of sight is not universal, with visual impairments posing significant challenges worldwide, particularly in countries like Bangladesh. The human eye, a small yet complex organ, is prone to various illnesses and conditions [1]. In clinical practice, assessing visual acuity (VA) status is a vital preliminary step, enabling eye care practitioners to detect ocular abnormalities or refractive errors [2]. VA gauges the eye's capacity to discern the shapes and intricate details of objects from a set distance [2]. It is essential to evaluate visual acuity consistently to identify any shifts in vision quality. Each eye is examined individually to ensure accuracy in the assessment. This is crucial from infancy onwards, as regular eye exams and vision tests play a key role in the effective treatment of visual impairments, thereby preventing blindness.

Globally, uncorrected refractive errors, cataracts, age-related macular degeneration (AMD), and glaucoma are the leading causes of visual impairment, with an estimated 80% of all vision problems being preventable [3]. In Bangladesh, the situation is dire. Research indicates that each year, approximately 150,000 individuals become blind, representing about 1.53% of the country's population [4]. Cataracts, easily treatable with simple surgery, account for the majority of these cases. However, the lack of awareness and healthcare access in Bangladesh exacerbates the problem.

The World Health Organization (WHO) estimates that globally, there are 284 million visually impaired individuals and 39 million blind people, with about 90% residing in low- and middle-income countries (LMICs) [5]. In these regions, a shortage of eye care professionals complicates the diagnosis, monitoring, and treatment of visual disorders. The National Eye Centre of Bangladesh's recent study reveals that in the adult population aged 40 and above, the prevalence of blindness is 1.0%, with higher rates in rural areas and among women [3]. These findings highlight the urgent need for innovative and accessible solutions.

The advent of mobile health (mHealth) technology offers a promising avenue to address the challenges in healthcare delivery and patient education, especially in developing and low- and middle-income countries like Bangladesh [6], [7], [8], [9]. In Bangladesh, as of June 2023, with an estimated 130 million smartphone and Internet users [10], mHealth has the potential to revolutionize eye care awareness and accessibility. Several studies have explored the development and application of mHealth in eye care. Abur Rahman et al. [11] introduced a VA test algorithm for mobile apps, using a single letter E display as the optotype. This innovation automatically generates results based on the WHO classification for vision impairment and blindness, marking a shift towards automated, user-friendly diagnostic tools. However, it requires further comparison with traditional methods for validation. Similarly, Luo et al. [12] investigated the use of a smartphone app for myopia screening, measuring refractive error by the far point distance. While innovative, the applicability of this approach remains to be thoroughly examined, especially in diverse, low-resource settings.

Further studies have compared mHealth solutions to traditional eye care methods. Rowe et al. [13] evaluated the vision screening assessment (VISA) test against professional vision evaluations in a prospective cohort study. This research highlighted the practicality of mHealth in clinical settings, though it could benefit from a broader demographic scope for enhanced generalizability. Stoll et al. [14] targeted pediatric populations, comparing the electronic measurement of VA (eMOVA) test with traditional exams like the Rossano-Weiss test. Their findings underscored the efficiency and child-friendliness of mHealth tools, a crucial aspect for countries like Bangladesh where child healthcare is paramount.

The Peek Community Screening app, developed by Rono et al. [15], integrated factors like age, vision, and pain into its referral algorithm, using the app as a reference standard. This study offered practical insights into the app's real-world utility. The same team, in another study [16], compared the Peek School Eye Health system to standard care, evaluating the app's accuracy and usability against the early treatment diabetic retinopathy study (ETDRS) LogMAR VA test chart. Peek employs a well established method in which a single optotype—the letter “E”—is shown in several ways. To test participants' visual acuity, they are asked to point out where the “E” is located. This approach works well in varied communities and places with varying reading levels since it is randomized and removes linguistic barriers. On the other hand, our method of vision testing is customized to the local environment and measures visual acuity using terms that are familiar to us. This enables us to guarantee that the test is more relatable for individuals who know the local language and engage participants in a way that is familiar to them culturally.

Validation and usability of mHealth tools have also been a focus. Rhiu et al. [17] created a VA testing app for the iPad, assessing its agreement with standard clinical tests. Their emphasis on user-friendliness and accuracy is crucial, though the app in less tech-savvy populations needs exploration. Venecia et al. [18] conducted a validation study of the Peek Acuity mobile app in pediatric populations in Paraguay, comparing its utility and economic viability against conventional methods, offering insights applicable to settings like Bangladesh.

Collectively, these studies highlight the integration of mobile technology in vision health, emphasizing the potential of mHealth solutions to enhance accessibility and efficiency in eye care. Yet, there is a gap in research specific to low-resource settings, where challenges like limited healthcare infrastructure and digital literacy are prevalent. To fill this gap, a mHealth app named “*Dristi*” was particularly developed to improve access to eye care services in Bangladesh. The application features tests for VA using Bengali letters to cater to the local population and also includes color blindness tests, eye health advice, and eye exercises. However, in this article, our investigation centers on the assessment of the *Dristi* application, specifically evaluating its performance in VA testing. The diagnoses provided by the application were meticulously compared with the ground truth, which consists of evaluations conducted by certified eye specialists.

In response to the urgent need for accessible eye care solutions and aligning with the WHO's advocacy for increased access to eye care services, our study contributes to the growing body of research on mHealth apps in eye care, particularly in LMICs like Bangladesh.

## 2 *Dristi* app design and development

In developing *Dristi* mobile app, we took inspiration from the Snellen chart [19], created in 1861, which utilizes abstract symbols based on a 5x5 unit grid. The original chart displays symbols such as A, B, C, E, G, L, N, P, R, T, 5, V, and Z. To enhance user-friendliness for our Bengali-speaking users, we adapted the chart to include Bengali letters like , , , , and  in place of the English letters.

We employed Craig's ophthalmology calculation [20] technique to compute eyeglass power based on the 20/20 standard. According to the Snellen chart [19] principles, patients must be presented with symbols from a distance of 20 feet or more to obtain accurate results. However, the development of a mobile app introduces constraints related to the size of mobile devices and the screen distance for displaying symbols or letters. Consequently, we opted for a standard visual distance of 2 feet to determine the appropriate letter sizes for the mobile app.

The design of letters on a vision chart is based on the concept of one minute of arc, which is the smallest angle of separation between two lines that the average human eye can discern. One minute of arc is equivalent to 1/60th of a degree. In vision testing, a letter is designed to encompass the smallest discernible space, comprising three bars and two spaces. The total height of a 20/20 letter thus equates to 5 minutes, with a 1-minute separation between the lines.

**Figure 1:**
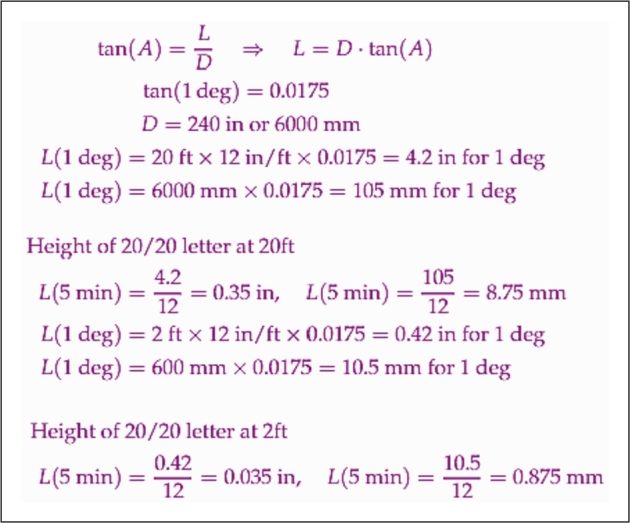
Calculation of letter size at the testing distance.

For calculating the size of letters at our testing distance, we utilized the concept of the Reduced Schematic Eye [21]. This model is a simplified representation of the eye's optics, instrumental in determining the sizes of objects and retinal images. Using the formula depicted in Fig. 1, we calculated the letter height, L, at a distance, D, for both the standard distances of 20 feet and 6 meters, as well as our app-specific distance of 2 feet. To simplify these calculations, we multiplied the result by 12 and divided it by 1 degree to convert the value into terms of 5 minutes.

Our mobile app, designed for Android devices, primarily functions to ‘Test the eye power.’ In addition to this major feature, the app includes a color blindness test, eye health advice, and eye exercise suggestions. In this study, our focus is exclusively on evaluating the performance of the *Dristi*'s features for distant and near vision testing.

**Figure 2:**
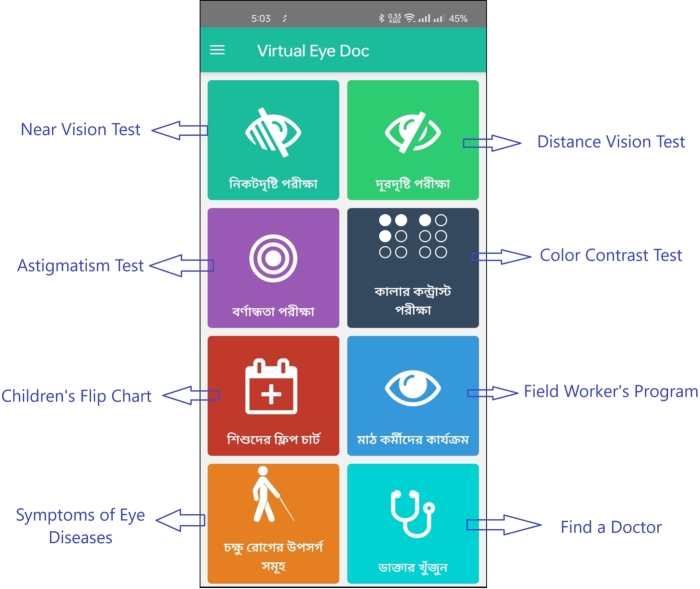
Homepage of *Dristi* app where the user can select different features.

**Figure 3:**
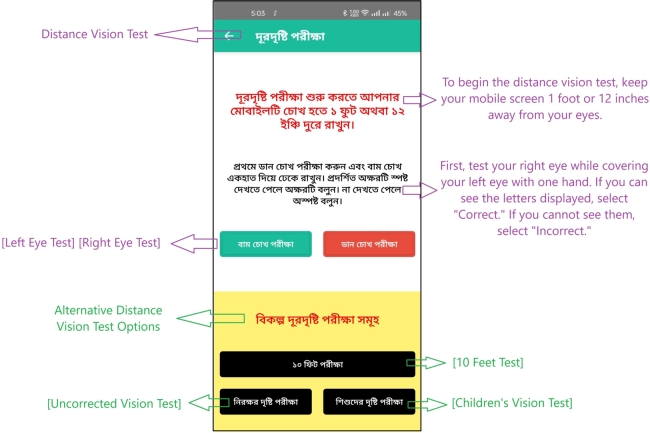
Instruction to proceed for an eye examination (distant vision test).

Upon logging into the app, the user is presented with a page offering various options, as depicted in Fig. 2. The next step involves detailed instructions on how to perform the eye test, including the precise distance at which the mobile phone should be held from the eye, shown in Fig. 3 for distant vision testing and Fig. 5 for near vision testing. The app then prompts the user to choose either the left or right eye for testing and instructs to close the other eye.

**Figure 4:**
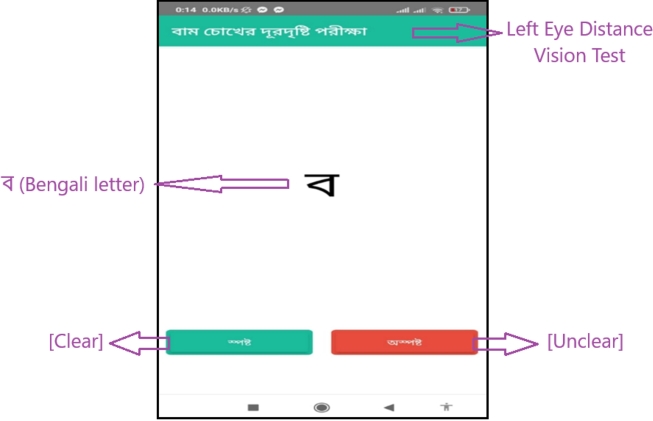
Visual acuity test of the left eye with a Bengali letter (distant vision test).

The test itself utilizes Bengali letters and text, as seen in the design of the subsequent pages, displayed in Fig. 4 for the distant vision test and Fig. 6 for the near vision test. To proceed with the test, the user clicks on “” (which means “Clear” in Bengali), leading to a page with 9 consecutive letters. If the letters appear blurry, the user can indicate this by pressing “” (meaning “Blur” in Bengali). Based on these interactions, the app calculates and displays the final result on the screen, as illustrated in Fig. 7.

**Figure 5:**
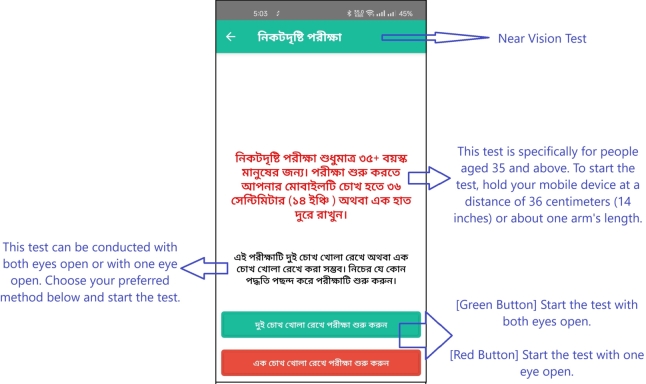
Instruction to proceed for an eye examination (near vision test).

**Figure 6:**
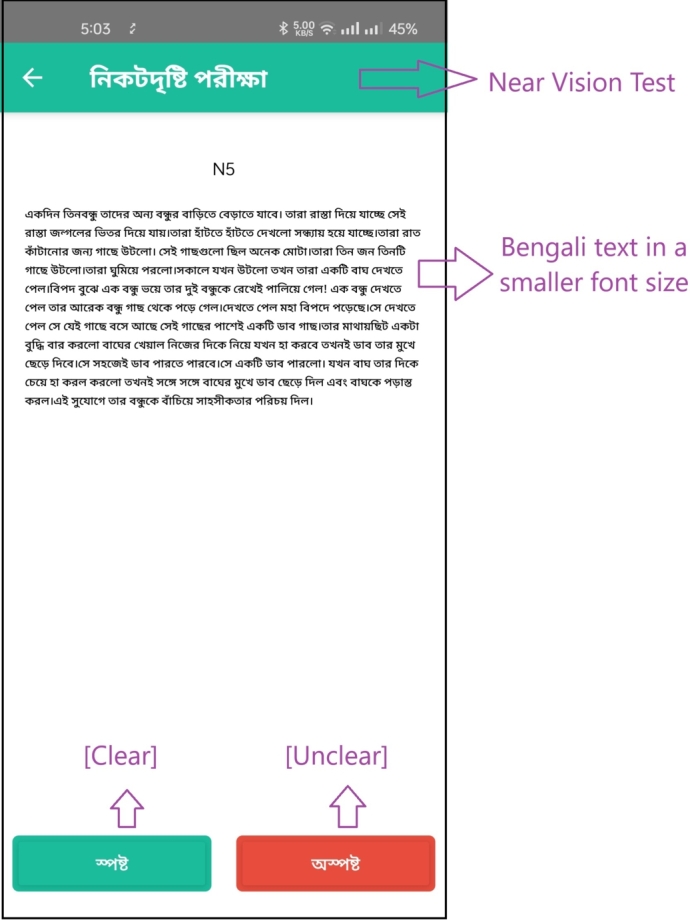
Visual acuity test with a Bengali text (near vision test).

**Figure 7:**
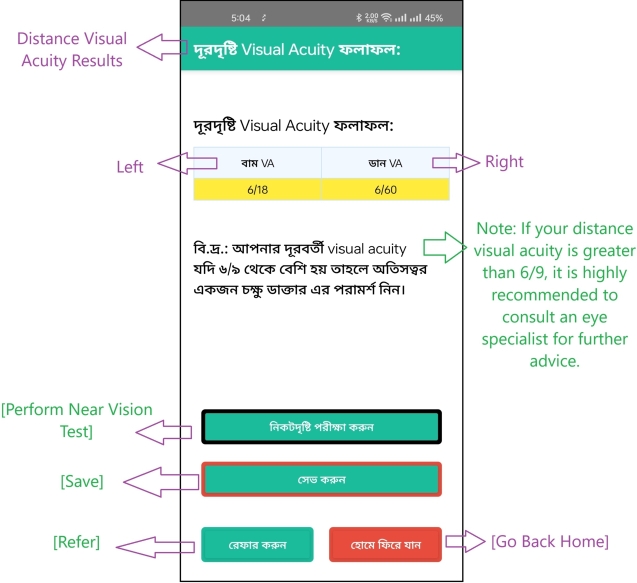
Explanation of result after finishing visual examination or visual acuity test.

## 3 Validation study methodology

### 3.1 Ethics

The Bangladesh Medical Research Council (BMRC) has given us the go-ahead to undertake this study in accordance with ethical standards. The study's goal was explicitly disclosed at the outset, and participants voluntarily gave their informed consent to participate. They were free to exit the study whenever they wanted. There were no predicted financial costs or incentives for participants. The consent of the participants, who were minors, was obtained from their guardians.

### 3.2 Study design

Between June 2022 and September 2022, a cross-sectional comparative design was employed for the clinical validation of *Dristi*. Before the eye test, the study was briefly explained, and informed consent was obtained. A standard Android smartphone device with good quality screen was used for the screening process in which the app was installed. The required demographic information, such as gender, age range, education level, occupation, and other health issues and symptoms, were requested from the participants. The demographics portion was followed by study tasks for the participants. They were first shown the screening process before having their distance vision checked while viewing a letter on a smartphone screen. We stopped screening that eye when they said there was a blur for letters of any size. We checked the other eye using the same method. Following that, we performed a near-vision screening. It took 10 to 15 minutes to finish each assignment.

We used the direction of the “E” sign to assess participants' visual status in order to overcome the difficulty of testing illiterate subjects in the study. We were able to assess distance vision using this method without having to depend on the participants' reading comprehension. Healthcare professionals were important in helping all participants comprehend and give appropriate answers by demonstrating the process to them. By using this method, the effect of illiteracy on the precision of our findings for distant vision was reduced.

**Figure 8:**
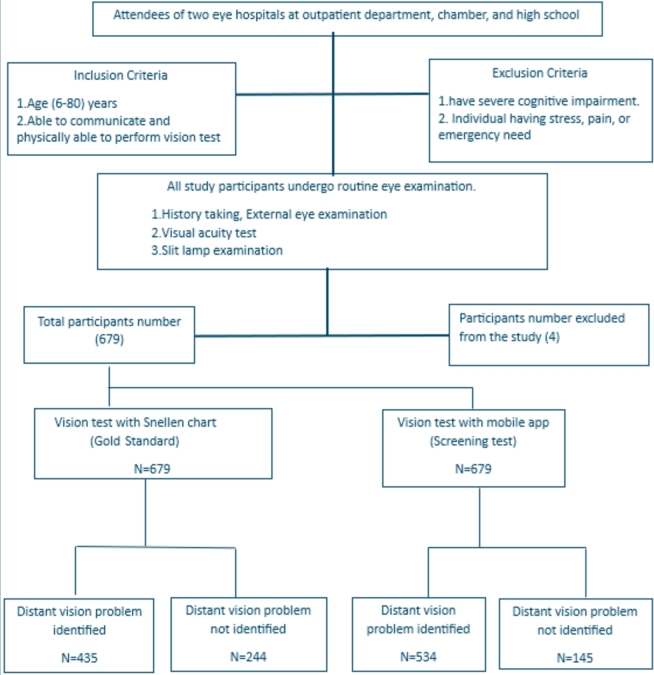
Flow diagram of procedures followed for distant vision testing among the participants.

**Figure 9:**
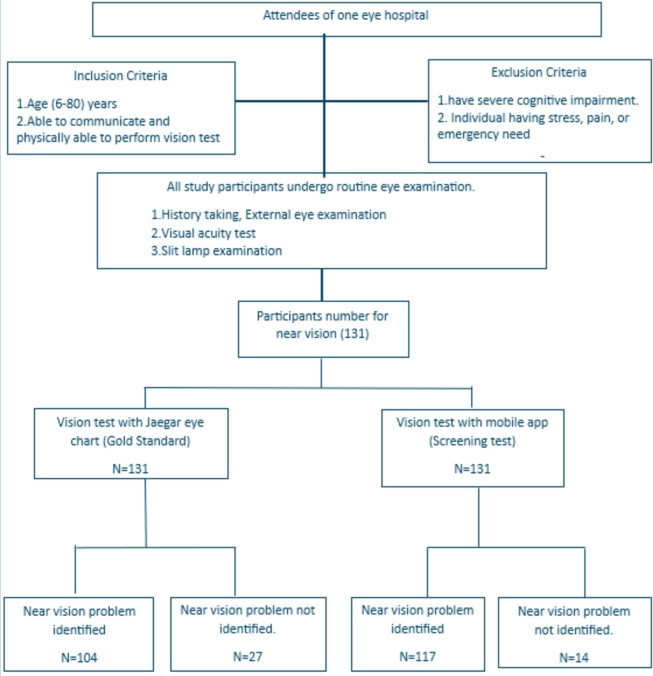
Flow diagram of procedures followed for near vision testing among the participants.

Fig. 8 and Fig. 9 illustrate the flow diagram of procedures followed during distant vision testing and near vision testing, respectively.

### 3.3 Participants recruitment

Individuals were suitable for inclusion if they were between 6 years to 80 years of age and had the ability to agree to vision screening using verbal or non-verbal indicators of agreement. Additional criteria were those who did not have cognitive impairment preventing screening, defined as difficulty with memory/concentration/decision making and thus being unable to follow instructions, and who did not decline vision screening. This was a convenience sampling of participants who were identified as being eligible by the outpatient eye department of related hospitals. Patients who had eye problems and had visited for a routine checkup were included in the study. Participants were asked about their history of additional co-morbid conditions like hypertension and diabetes mellitus, which are highly typical causes of difficulties for the eyes.

A required minimum sample size of 219 individuals was calculated using Burderer's formula [22] in Equation (1). For the calculation, we assumed a prevalence of 63.2% for the refractive error eye diseases in the Bangladeshi population [23] whereas we anticipated a sensitivity of 90% and a confidence interval (CI) of 95%.

(1) $$n=\frac{Z_{1-\alpha /2}^{2}\times \text{SN}\times (1-\text{SN})}{L^{2}\times \text{Prevalence}}$$

Where:

- *n* = sample size

- SN = anticipated sensitivity = 0.90

- $Z_{1-\alpha /2}=1.96$ (Standard normal deviate at 95% CI)

- L=0.05 (allowable error), and

- Prevalence = 0.632

### 3.4 Data collection

We have collected data from two eye hospitals, one private clinic, and one high school. One tertiary hospital was the government's special eye hospital and training center situated in Gopalganj, where patients from the south region of Bangladesh come for various vision problems. Another semi-private hospital is situated in Dhaka, the center of Bangladesh, where patients with vision problems come from across the country to get better service. Data has also been collected from one private clinic in Dhaka city where patients get care intensively for their eyes. For this study, trained data collectors working under an eye doctor's supervision used the *Dristi* app to conduct vision screenings. They recorded the clinician's VA results for each patient in both hard copy and as an image on a mobile phone. Both distant and near vision were screened to validate the *Dristi* app by comparing its results with those obtained by the clinician.

### 3.5 Performance metrics

*Dristi*'s performance in terms of VA testing was measured through findings of true positive (TP), false positive (FP), true negative (TN), and false negative (FN) cases. Based on these data, accuracy, sensitivity, specificity, positive predictive value (PPV), and negative predictive value (NPV) were calculated according to Equations (2), (3), (4), (5), and (6).

(2) $$\text{Accuracy}=\frac{TP+TN}{TP+TN+FP+FN}$$

(3) $$\text{Sensitivity}=\frac{TP}{TP+FN}$$

(4) $$\text{Specificity}=\frac{TN}{TN+FP}$$

(5) $$\text{PPV}=\frac{TP}{TP+FP}$$

(6) $$\text{NPV}=\frac{TN}{TN+FN}$$

### 3.6 Data analysis

#### 3.6.1 Descriptive analysis

Microsoft Excel was used to document and analyze the data. After documentation, the data were cleaned by two researchers. The descriptive analysis of the closed questions, which included the calculation of means, standard deviations (SDs), percentages, and frequency distributions, was done in Excel. Other data analyses, such as sensitivity, specificity, positive predictive value, negative predictive value, and accuracy of the app, were calculated using a confusion matrix. The researchers compared the app's findings with those of the clinicians. Data were captured accurately by observing the photographs of the clinicians' prescriptions, the hard copies from the data collectors, and the documents from the *Dristi* app.

#### 3.6.2 Statistical analysis

To assess whether the app's performance for distant vision testing was consistent across various settings (Government Eye Hospital, Semi-Private Eye Hospital, Private Clinic, and High School), a Chi-square test [24] was carried out to examine potential heterogeneity across these sites. This analysis aimed to determine whether the app's performance remained statistically uniform or if there were notable discrepancies requiring further investigation. The hypotheses formulated for the Chi-square test were as follows:

• **Null Hypothesis (H**_**0**_**):** The performance of the app (in terms of true positives, true negatives, false positives, and false negatives) is consistent across different settings (Government Eye Hospital, Semi-Private Eye Hospital, Private Clinic, and High School). There is no significant difference in the proportions of categories between the groups. The distributions are homogeneous.
• **Alternate Hypothesis (H**_**1**_**):** The performance of the app varies across different settings. There is a significant difference in the proportions of categories between the groups. The distributions are heterogeneous.

## 4 Results analysis

### 4.1 Participants

Initially, 683 (male = 297, female = 386) individuals having a mean age of 35.4 years (12 to 80 years range) and an SD of 16.10 agreed to participate. After screening their eyes with the app, reports were cross-checked with doctors' reports by taking a photograph of their prescription. Among 683 participants, 4 participants denied taking photographs of their prescription. So, four people were excluded from the study. After the exclusion of these missing data, one-to-one cross-checking was done by the research team. Finally, a total of 679 people's data was included in the study (male = 297, female = 382), ranging in age from 12 to 80 years (mean = 35, SD = 16.30). Table 1 presents the overview of the demographics of the participants.

**Table 1:** Demographics of Participants (n=679).

			Count (n)	Percentage (%)
Gender				
	Male		297	43.74
	Female		382	56.26
Residency				
	Rural		356	52.43
		Male	130	36.52
		Female	226	63.48
	Urban		323	47.57
		Male	167	51.70
		Female	156	48.30
Education				
	Illiterate		127	18.70
	Primary		241	35.49
	Secondary		152	22.39
	Higher Secondary		102	15.02
	Graduate		57	8.39
Occupation				
	Private Job		73	10.75
	Government Job		23	3.39
	Business Owner		57	8.39
	Student		127	18.70
	Homemaker		221	32.55
	Others		178	26.22
Health Condition				
	Diabetic		88	12.88
	Hypertension		170	24.89
	Other Associated Diseases		162	23.72
	No Health Issues		259	38.14

Moreover, we identified 162 individuals among 679 individuals who had attended the hospital with other related issues, such as red eyes, itchy eyes, blurred vision, cataracts, painful eyes, and allergies. In our investigation, we also discovered patients with diabetes and hypertension who are more likely to experience eye problems. Among the 162 individuals, we identified that refractive error affects 56 of them.

### 4.2 Performance evaluation

From the study on distant vision, 411 data were identified as true positive whereas 121 data were true negative. On the other hand, 123 data were false positive, and 24 data were false negative. Table 2 and Table 3 present the performance evaluation metrics and findings for distant vision testing and near vision testing by *Dristi* app, respectively.

**Table 2:** Performance analysis of *Dristi* across four different settings for distant vision (Rounding off numbers to two decimal places).

	Government Eye Hospital	Semi-Private Eye Hospital	Private Clinic	High School	Total
True Positive	193	123	85	10	411
True Negative	26	34	8	53	121
False Negative	8	11	2	3	24
False Positive	27	56	4	36	123

Total	254	224	99	102	679

Accuracy	86%	70%	94%	62%	
Sensitivity	96%	92%	98%	77%	
Specificity	49%	38%	67%	60%

**Table 3:** Performance analysis of *Dristi* at the semi-private eye hospital for near vision (Rounding off numbers to two decimal places).

	Semi-Private Eye Hospital
True Positive	100
True Negative	10
False Negative	4
False Positive	17

Total	131

Accuracy	84%
Sensitivity	96%
Specificity	37%

As data were collected from four sources for distant vision testing, the accuracy of *Dristi* ranges from (62-86)% with sensitivity ranges from (77-98)%, and specificity ranges from (38-67)% for distant vision testing.

Near vision testing data were gathered from only one source and we could test 131 people's eyes for near vision whose ages were above 35 years. For near vision, we have an accuracy of 84% with a sensitivity of 96%, and a specificity of 37%.

**Figure 10:**
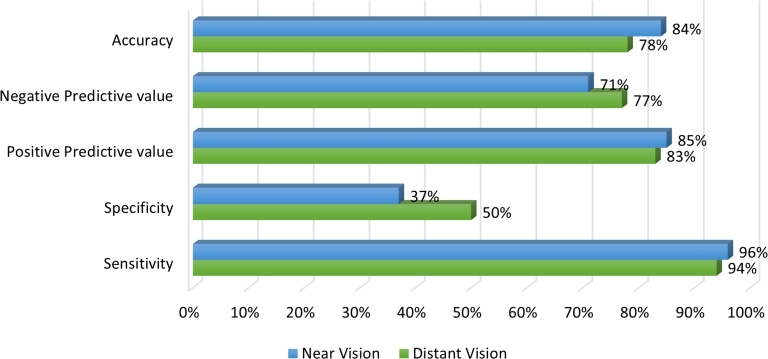
Comparative performance analysis of *Dristi* for both distant vision testing (average from 4 data sources) and near vision testing.

Fig. 10 illustrates the comparative analysis of *Dristi*'s performance for both distant vision testing and near vision testing. The averages of all the performance indicators for 4 data sources are presented here for the distant vision testing only.

#### 4.2.1 Distant vision testing

At the government eye hospital, the app registered a high number of true positives (n=193) which is indicative of its effectiveness in a clinical setting with a potentially higher prevalence of vision issues. However, it also showed a relatively lower specificity (49%), meaning there were quite a few false positives. This could suggest that in a hospital setting, where patients might already have known eye issues, the app is erring on the side of caution, perhaps to avoid missing any potential diagnoses.

The semi-private eye hospital saw a lower accuracy (70%) compared to the government eye hospital. With the highest number of false positives (56), it seems that the app may be overly sensitive in this environment. The sensitivity is still high at 92%, indicating that the app is adept at correctly identifying cases that are indeed positive.

The private clinic showed remarkable accuracy (94%) and the highest sensitivity (98%). The app seems to perform at its best in this setting, reliably identifying those with vision issues. The specificity is also relatively high at 67%, suggesting a good balance between identifying true positives and avoiding false alarms.

The high school setting presented the lowest accuracy (62%) and sensitivity (77%), which might reflect a lower occurrence of vision issues among a younger population or potential underutilization or ineffectiveness of the app in this context. Nevertheless, the specificity is higher than in the semi-private eye hospital (60%), meaning that when the app identifies a student as not having vision issues, it is more often correct than not.

The overall negative predictive value of 83% across all settings indicates that when the app predicts a negative result, it is likely to be correct 83% of the time. On the other hand, the positive predictive value of 77% suggests that positive results need to be considered more cautiously, as approximately 1 in 4 positive results may not represent a true vision issue.

Overall, across all locations, the app's performance varied, with the best results seen in more controlled environments like the private clinic. A notable pattern is the variation in specificity, which suggests that the app's algorithm might be fine-tuned to improve its ability to correctly identify when vision issues are not present, thereby reducing the rate of false positives. The differences in performance metrics across locations underline the importance of considering the context in which such apps are deployed. Factors such as the demographic of the tested population, the prevalence of vision issues, and the setting's conditions may influence the results.

#### 4.2.2 Near vision testing

The evaluation of the near vision testing capabilities of the app at the semi-private eye hospital yielded promising results, yet it also highlighted areas for potential refinement. The app achieved a high number of true positives (n=100), demonstrating its effectiveness in identifying near vision issues with a high degree of accuracy, as reflected in the sensitivity score of 96%. This suggests that the app is highly capable of detecting genuine vision problems when they are present.

Despite these encouraging indicators, the specificity was relatively low at 37%, indicating a considerable number of false positives (n=17). This could imply that the app may be prone to over-diagnosing patients in this particular setting, which could lead to unnecessary further testing or unwarranted concerns for patients. The specificity metric here underscores the need for the app's diagnostic criteria to be potentially adjusted to better distinguish between true and false positives in near vision testing scenarios.

Interestingly, the accuracy of the app was relatively high at 84%, which points to a robust overall performance. However, the specificity figure must be taken into account, as it tempers the interpretation of the app's accuracy. The True Negative count (n=10) while lower than the True Positive, confirms that when the app does categorize a test result as negative, it is usually correct, supported by a negative predictive value of 83%.

The positive predictive value of 77% suggests that there is still room for improvement in ensuring that positive test results are true positives. About 1 in 4 individuals receiving a positive result may not have a near vision problem, which could lead to over-referrals.

The data points to a need for a balanced approach in the app's algorithm, which should be sensitive enough to detect real issues (as evidenced by the high sensitivity) but also discerning enough not to flag too many false positives, which is especially important in environments like a semi-private eye hospital where patients expect accurate and reliable screening results.

### 4.3 Statistical analysis for heterogeneity: distant vision testing

The Chi-square test for Heterogeneity yielded a test statistic of 189.32 with 9 degrees of freedom and an associated p-value of 5.72e-36. Given that the p-value is significantly lower than the conventional threshold of 0.05 for statistical significance, we reject the null hypothesis (H_0_)). This finding suggests that the app's performance across the different locations is not statistically consistent, indicating significant heterogeneity among the sites. The results reveal that there are notable differences in the distribution of outcomes (true positives, true negatives, false positives, and false negatives) across the various testing locations, which included Government Eye Hospital, Semi-Private Eye Hospital, Private Clinic, and High School. The assumption of homogeneity is violated, implying that the app's performance varies across these settings.

The heterogeneity observed across these locations suggests that contextual factors unique to each site may be influencing the app's outcomes. Several possible explanations could account for this variation. First, demographic differences such as age, socioeconomic status, and prevalence of vision-related conditions may vary across locations, affecting both the patient population and the types of vision challenges encountered. Second, operational factors such as the quality of equipment, the proficiency of healthcare workers, or differences in number of patients attending may also contribute to this observed heterogeneity. Environmental conditions such as lighting, space constraints, and overall clinical environment could further influence the testing process and outcomes.

### 4.4 Analysis of sensitivity and specificity across different settings

#### 4.4.1 Distant vision testing

The accuracy metrics, specifically sensitivity and specificity, were recalculated for each testing location, and their corresponding 95% CIs were computed to provide a deeper understanding of the app's performance reliability. The results are presented in Table 4.

**Table 4:** Sensitivity and specificity with 95% CIs across different locations/settings for distant vision testing.

Location	Sensitivity (95% CI)	Specificity (95% CI)
Government Eye Hospital	96.0% (92.34% to 97.97%)	49.1% (36.12% to 62.12%)
Semi-Private Eye Hospital	91.8% (85.90% to 95.35%)	37.8% (28.46% to 48.10%)
Private Clinic	97.7% (92.00% to 99.37%)	66.7% (39.06% to 86.19%)
High School	76.9% (49.74% to 91.82%)	59.6% (49.16% to 69.15%)

The sensitivity results across the locations are generally high, indicating that the app performs well in detecting true positives. However, the specificity values show a much wider variation, particularly at the Government and Semi-Private Eye Hospitals, where specificity is notably low. This suggests a higher occurrence of false positives at these sites, which may point to inconsistencies in diagnostic accuracy across different settings.

The broad CIs, particularly for specificity in certain settings, reflect variability and uncertainty in the app's ability to consistently identify true negatives. These variations underline the need for further investigation into location-specific factors, such as differences in patient demographics, testing environments, or the number of patients attending, which may be influencing the app's performance. Identifying and addressing these factors will be crucial for improving the app's reliability across diverse healthcare settings.

#### 4.4.2 Near vision testing

Based on the data from the Semi-Private Eye Hospital for near vision testing, the app's performance metrics were recalculated, including their 95% CIs to assess reliability. The results are presented in Table 5. The high sensitivity, along with its narrow confidence interval, suggests consistent performance in identifying true positives. However, the specificity is notably lower, with a broader confidence interval, indicating a potential variability in detecting true negatives. This lower specificity points to a higher rate of false positives, which may require further investigation to understand the underlying factors contributing to this inconsistency in the app's performance.

**Table 5:** Sensitivity and specificity with 95% CIs for near vision testing.

Location	Sensitivity (95% CI)	Specificity (95% CI)
Semi-Private Eye Hospital	96.2% (90.53% to 98.49%)	37.0% (21.53% to 55.77%)

## 5 Discussion

### 5.1 Principal findings

The goal of the study was to support the development of the *Dristi* app, which was meant to be used for the initial diagnosis of visual issues. It has been demonstrated that this mHealth app can lessen eyesight issues in LMICs like Bangladesh where there are not enough ophthalmologists in the outlying areas. It can test the primary VA and recommend them to an eye care facility nearby. Other aspects of the *Dristi* platform include tests for color blindness and color contrast, eye exercises, and signs of common eye conditions. All of the instructions and information in these parts are provided in Bangla, making them accessible to the nation's disadvantaged groups. They have quick access to general knowledge about the eye. Before being added to the digital portal, ophthalmologists verified all of the information.

In assessing the efficacy of the distant vision testing application, our findings draw from an array of healthcare environments, each offering unique insights into the app's diagnostic strengths and areas ripe for enhancement. At the government hospital, the app's high detection rate of true positive cases signals its strong potential in clinical settings, where such issues are presumably more common. Yet, this same setting revealed a tendency for the app to produce false positives, suggesting a cautious approach in its diagnostic process, which, while erring on the side of caution, could lead to unnecessary further evaluations.

In contrast, the semi-private eye hospital's data tell a story of high sensitivity overshadowed by a higher rate of false positives, hinting at an over-sensitive diagnostic criterion. This sensitivity ensures that few cases slip through the net but at the cost of potential over-diagnosis. The app's performance in private clinics was notably superior, showcasing high accuracy and sensitivity. This suggests that the app thrives in well-regulated environments, offering a reliable resource for initial vision screening. The scenario at the high school differed, marked by the lowest accuracy and sensitivity, possibly pointing to the app's underutilization or a mismatch between the app's design and the younger demographic's needs. However, the higher specificity here reassures us of the app's ability to accurately rule out vision problems in students.

Collectively, these environments, characterized by a reasonable negative predictive value and a positive predictive value that invites a careful interpretation, affirm the app as a valuable preliminary screening tool. Nonetheless, the varied performance metrics underscore the importance of tailoring the app to each environment's unique context. This includes adapting to demographic differences, the prevalence of vision conditions, and the particularities of each testing location. In essence, while the app has proven its merit in aiding early vision impairment detection, the data implore us to apply its results with discernment, taking into account the distinct characteristics of each testing ground. Such a considerate application will ensure the app's role not just in screening but as an integral part of a holistic eye care strategy.

### 5.2 Limitations and challenges

In Bangladesh, a nation where English is not the native language of the populace, the adoption of English-language-based mobile apps is usually unexpected. Consequently, the *Dristi* app was conscientiously developed in Bengali to better serve the predominantly rural communities. Despite the app's user-friendly design, several limitations emerged during the pilot phase.

During sociodemographic data collection, people could not mention their date of birth, phone number, or address properly. Due to illiteracy, a communication gap was identified during screening with the *Dristi* app. Due to an electricity shortage, load shedding was frequent in the hospitals, which hampered the data collection process. The app requires an Internet connection to complete the screening procedure, which has limited our app's activities.

The substantial patient volume in hospitals also posed challenges, occasionally compromising the quality of service and, by extension, the data collection process. Privacy concerns were another barrier, as some patients were reticent to permit photographs of their prescriptions, necessitating the exclusion of certain data points from our dataset.

A notable discrepancy was observed when the app was used to screen individuals who had recently undergone surgical procedures. While clinical assessments by doctors indicated perfect vision (6/6), the app sometimes detected refractive errors. This inconsistency underscores the need for further calibration of the app to ensure its diagnostic accuracy aligns with professional medical evaluations.

Moreover, our study was limited by the use of near-vision tests, especially for the few illiterate participants. Illiteracy might have affected the outcomes in these instances since the “E” sign is less useful for near-vision evaluations.

### 5.3 Comparison to prior work

There are several types of visual acuity (VA) tests commonly used in various settings. The Standard Snellen Chart [19], a well-known test, requires individuals to read letters from a chart positioned 20 feet away (n=24). The LogMAR Chart is another standardized method that measures acuity using the “Logarithm of the Minimum Angle of Resolution” and is often used in research settings [25]. The Tumbling E or Landolt C Chart is particularly helpful for individuals who cannot read or speak the language, involving the identification of the orientation of an “E” or a broken ring [26].

Our VA testing app innovatively incorporates selected Bengali letters such as , , , , and , distinguishing it from typical apps that often employ the entire alphabet or a standard set of symbols and letters. This approach ensures wider applicability, sometimes extending options for multiple languages or scripts for diverse users. Our app tests each eye individually, unlike many basic apps that test both eyes together, which can miss differences between the eyes. This method can help identify issues that might otherwise be overlooked.

Additionally, the app's emphasis on a fixed distance primarily measures near vision, a departure from many competitors that allow for both near and distance vision evaluations by adjusting testing distances or letter dimensions. This offers a more comprehensive view of a person's visual faculties. With regard to specialized treatment, the app goes beyond simple acuity testing by providing eye health recommendations, indicating a commitment to holistic eye wellness rather than focusing solely on specific problems like diabetic retinopathy. This contrasts with some specialist apps created especially for crucial issues such as diabetic retinopathy, which are essential in continuous diabetes management.

## 6 Conclusion and future directions

This paper presented a novel mHealth app designed to facilitate eye screening and promote eye health awareness in Bangladesh. Tailored for Android devices, our app integrates essential features such as VA testing using Bengali letters, color blindness tests, and valuable eye health and exercise advice. This initiative represents a significant step towards making eye care accessible and user-friendly, particularly in a country where healthcare resources are limited and digital technology is rapidly expanding. Based on the findings, several future directions for this work can be:

• Expanding Accessibility and Compatibility: Future iterations of the app should aim for cross-platform compatibility, including iOS systems, to expand its reach. Additionally, integrating voice commands and audio feedback can enhance accessibility for users with limited literacy or tech-savviness.
• User Experience and Interface Improvements: Continuous improvement in user interface design is crucial. Future versions could include more intuitive navigation, personalized settings, and multi-lingual support to cater to the diverse population of Bangladesh.
• Advanced Diagnostic Features: Incorporating advanced diagnostic features, such as automated detection of eye diseases using artificial intelligence, can significantly enhance the app's utility. This advancement would require extensive research and validation but has the potential to revolutionize early eye disease detection.
• Telemedicine Integration: Incorporating telemedicine features where users can consult with eye care professionals directly through the app could be a game-changer, especially in remote areas with limited access to specialists.
• Data Analytics for Public Health Insights: Collecting and analyzing user data (while respecting privacy and ethical considerations) could provide valuable insights into the prevalence of eye conditions in different demographics and regions, aiding public health planning and interventions.
• Scalability to Other Regions: Once established in Bangladesh, there's potential for adapting and scaling the app to other similar low-resource settings, taking into account local languages, cultural nuances, and healthcare infrastructures.
• Robust Clinical Trials and Research: Conducting robust clinical trials to assess the app's efficacy and safety will be crucial. This research would provide the necessary evidence to support wider adoption and continuous improvement.
• Feedback Mechanisms and Community Engagement: Implementing user feedback mechanisms to continuously refine the app based on user experiences and preferences will be essential. Engaging with the community through workshops and awareness campaigns can also enhance the app's adoption and impact.
• Cross-Platform Development: In future work, the ‘Dristi’ app, which was initially developed for Android OS due to its widespread use in Bangladesh, will be expanded into a cross-platform app. This transformation will enable the app to be compatible with other commonly used operating systems, ensuring broader accessibility and usability across different devices.

In conclusion, while our mHealth app presents a promising solution to the challenges of eye care accessibility in Bangladesh, its true potential lies in continuous evolution and integration with broader healthcare initiatives. By addressing these future directions, the app can significantly contribute to the prevention of visual impairments and the promotion of eye health at a national and potentially global scale.

## Abbreviations

The following abbreviations are used in this manuscript:

**Table:** 

AMD	Age-related macular degeneration
BMRC	Bangladesh Medical Research Council
VA	Visual acuity
LMIC	Low- and Middle-income Country
WHO	World Health Organization
mHealth	Mobile Health

## Informed consent statement

Informed consent was collected from each of the participants.

## Ethics/institutional review board statement

Ethical approval was obtained from the Bangladesh Medical Research Council (BMRC), Bangladesh. The registration number at BMRC is 446 30 09 2021.

## Declaration of generative AI and AI-assisted technologies in the writing process

ChatGPT by OpenAI Inc. was used to rewrite some of the texts for better readability but no data or information was generated by it.

## Funding

This work was supported by the ICT Innovation Fund, Information and Communication Technology Division, Ministry of Posts, Telecommunications and Information Technology, Government of Bangladesh.

## CRediT authorship contribution statement

**Monzurul Haque:** Writing – review & editing, Writing – original draft, Software, Methodology, Formal analysis, Data curation. **Marzia Zaman:** Writing – review & editing, Writing – original draft, Validation, Methodology, Investigation, Formal analysis, Data curation. **Ashraful Islam:** Writing – review & editing, Validation, Supervision, Project administration, Investigation, Formal analysis. **Farhana Sarker:** Writing – review & editing, Validation, Supervision, Resources, Project administration, Investigation, Formal analysis. **Nahid Ferdausi:** Validation, Supervision, Resources, Project administration. **Khondaker A. Mamun:** Writing – review & editing, Supervision, Resources, Conceptualization, Project administration, Investigation, Funding acquisition, Data curation.

## Declaration of Competing Interest

The authors declare that they have no known competing financial interests or personal relationships that could have appeared to influence the work reported in this paper.

## Data Availability

The data collected during the user studies is not publicly available. However, it may be shared upon request.
